# Characteristics of hypoparathyroidism in Colombia: data from a single center in the city of Medellín

**DOI:** 10.20945/2359-3997000000250

**Published:** 2020-06-05

**Authors:** Julián Zea Lopera, Sergio Alberto Londoño Tabares, Daniela Álvarez Herrera, Esteban Cardona Henao, Fabian Alberto Jaimes Barragán, Carlos Alfonso Builes Barrera, Juan David Gómez Corrales, Catalina Rúa Marín, Diva Cristina Castro, Alejandro Román-González

**Affiliations:** 1 Universidad de Antioquia Medellín Colombia Universidad de Antioquia, Medellín, Colombia; 2 Universidad de Antioquia Hospital Universitario San Vicente Fundación Medellín Colombia Universidad de Antioquia; Hospital Universitario San Vicente Fundación, Medellín, Colombia; 3 Hospital Universitario San Vicente Fundación Medellin Colombia Hospital Universitario San Vicente Fundación, Medellin, Colombia

**Keywords:** Hypoparathyroidism, parathyroid, hypocalcemia, hyperphosphatemia, calciuria

## Abstract

**Objective:**

Hypoparathyroidism is a rare condition, whose most common etiology is complications of neck surgery. The aim of the study was to identify the clinical and biochemical profile of the patients with diagnosis of hypoparathyroidism, including the frequency of symptoms, clinical signs, long-term complications and disease control. Additionally, the study sought to know what the medication profile was, and the doses required by the patients.

**Subjects and method:**

A retrospective cohort study was conducted wherein all patients with ICD-10 codes associated with hypoparathyroidism between 2011 and 2018 at the Hospital Universitario San Vicente Fundación were included. We investigated the etiology of the disease; biochemical profile including lowest serum calcium, highest serum phosphorus, 25OHD levels, calciuria and calcium/phosphorus product; medication doses, disease control, and presence of complications, especially renal and neurologic complications were also evaluated.

**Results:**

The cohort included 108 patients (99 women/9 men) with a mean age of 51.6 ± 15.6 years. The main etiology was postoperative (93.5%), the dose of elemental calcium received was relatively low (mean 1,164 mg/day), and in only 9.2% of cases more than 2,500 mg/day of elemental calcium was necessary. We were able to evaluate the follow-up in 89 patients, and found that only 57.3% met the criteria for controlled disease.

**Conclusion:**

The clinical profile of patients with hypoparathyroidism in our cohort is similar to that described in other international studies, with predominantly postoperative etiology. With standard therapy, only adequate control is achieved in a little more than half of patients. Arch Endocrinol Metab. 2020;64(3):282-9

## INTRODUCTION

Hypoparathyroidism is characterized by low serum concentrations of parathyroid hormone (PTH), which results in hypocalcemia and hyperphosphatemia ( 1 , 2 ). In the United States, it is estimated that the prevalence of this condition is nearly 37 cases per 100,000 inhabitants, of which 8 cases per 100,000 have non-surgical etiology and 29 cases per 100,000 are postoperative ( 3 , 4 ). In European countries, a prevalence of similar proportions to that in the United States is described ( 5 , 6 ). The primary etiology is accidental surgical resection of the parathyroid glands during procedures such as thyroidectomies, but less frequent causes are also described, such as autoimmune diseases, infiltrative diseases, and neck radiation, among others ( 7 ). Clinical manifestations can be acute, such as neuromuscular irritability, weakness, seizures, and laryngospasm, or they can be chronic, affecting a large number of systems. The latter are explained by the persistence of an inadequate balance between serum calcium and phosphorus over time, which allows for the deposit of calcium in different tissues (calcification of the basal ganglia, nephrocalcinosis, etc.) ( 8 ). The main axis of management is the replacement of calcium and calcitriol, but in some selected cases hormonal replacement with PTH can be used ( 9 - 16 ).

Knowing the complete clinical profile of patients presenting with this disease, as well as having an estimate of the main clinical manifestations, physical findings, and complications, would allow us to have a broader view of the disease, which would ultimately result in earlier diagnosis, especially in groups that may be predisposed to developing the disease, such as patients who have undergone neck surgery. In addition, with the identification of clinical and laboratory variables, prediction models can be proposed for this disease, thus allowing for early diagnosis.

In Colombia, to the best of our knowledge, characterization of patients with hypoparathyroidism and determination of the etiology of the disease have not yet been performed, and the clinical manifestations have also not been established along with how often each one occurs. In addition, data in Latin America is limited. The objective of this study was to determine the clinical and paraclinical characteristics of patients with hypoparathyroidism who were treated at a high complexity hospital in one of the country’s main cities.

## SUBJECTS AND METHODS

A retrospective cohort study was conducted at a single tertiary care hospital in Medellín, Colombia and approved by the Ethics Committee (Hospital Universitario San Vicente Fundación – HUSVF/Universidad de Antioquia). Patients over 18 years of age who had low PTH concentrations (< 20 pg/mL) for more than 6 months and who had been evaluated at least once in the hospital for symptoms of hypocalcemia were included (including emergency consultations and outpatient care at the hospital). Patients under 18 years of age with pseudohypoparathyroidism and those who did not have a PTH report in their clinical history or laboratory tests were excluded.

We recorded the demographic variables (sex, age, and race); clinical aspects (etiology, hospital/outpatient management, previous diagnosis of hypoparathyroidism, time of evolution of symptoms, comorbidities, symptoms and signs associated with hypocalcemia, and chronic complications of the disease); biochemistry (lowest serum calcium, initial calcium, 24-h calciuria, phosphorus, 25OHD, albumin, and PTH); and associated disease management (elemental calcium dose, presentation of calcium used, calcitriol dose, thiazide diuretic use, PTH analog use, disease control, emergency consultations, and number of hospitalizations). This study was authorized by the HUSFV Ethics Committee.

### Definitions

Hypoparathyroidism was defined as a low PTH concentration (<20 pg/mL) accompanied by symptoms of hypocalcemia or the requirement for calcium or calcitriol supplementation to avoid their onset. It was necessary for patients to meet these criteria for more than 6 months, as a shorter time indicates the presence of transient hypoparathyroidism. This specific population was not taken into account in the present study as its complication profile is different from those with permanent hypoparathyroidism (they have no risk of chronic complications of the disease). Hypocalcemia was defined as a serum calcium concentration lower than 8.4 mg/dL, corrected for albumin.

Disease control was defined as minimal variation or stability in the result of serum calcium, phosphorus, and calciuria in at least two controls, at least 2 months apart. In cases where only the value of calcium was available, this parameter was used to define if the disease was controlled in patients who had undergone more than one assessment ( 9 , 11 , 17 ).

Indications for the use of PTH were taken from present international recommendations that include: elemental calcium dose >2.5 g/day, calcitriol dose >1.5 ug/day, calcium/phosphorus product >55 mg^2^/dL^2^, hypercalciuria (>250 mg/24 h in women and >300 mg/24 h in men) and presence of renal complications (nephrocalcinosis and nephrolithiasis) ( 11 ).

### Data collection

Authorization was obtained from the HSFV research department to access the database of patients treated between 2011 and 2017 who were associated with the following ICD-10 codes: D821 (DiGeorge syndrome), E200 (idiopathic hypoparathyroidism), E201 (pseudohypoparathyroidism), E208 (other types of hypoparathyroidism), E209 (unspecified hypoparathyroidism), E58X (dietary calcium deficiency), E835 (calcium metabolism disorder), E892 (hypoparathyroidism secondary to procedures). Data collection was performed between October 11, 2017 and November 9, 2018 by four researchers (JZL, SALT, DAH, and ECH) using a preset format with predefined variables, available on GoogleDocs^®^. Patients with an equivocal diagnosis were reviewed by two authors (JZL, SALT) who reached a diagnostic consensus.

### Statistical analysis

The statistical program SPSS Statistics 25 (IBM, Chicago) was used to analyze the data. Continuous variables are presented as medians and interquartile ranges, or as means and standard deviation according to the distribution of a given variable with the Shapiro-Wilk test. Categorical variables are reported as frequencies and percentages. The difference between median time was compared using non-parametric test mainly the Chi-squared test. To compare frequencies between groups a Pearson Chi-squared test was done.

## RESULTS

A total of 1,422 medical records were reviewed to which inclusion and exclusion criteria were applied. In the end, 108 patients were included in the study and 1,314 patients who did not meet inclusion criteria were excluded. The main reason of exclusion was the absence of serum PTH values (n = 368), followed by duplicate records (n = 192), children (n = 317), normal PTH (n = 103), hyperparathyroidism (n = 125), hypercalcemia (n = 191), transient hypoparathyroidism (n = 6), pseudohypoparathyroidism (n = 6), hypomagnesemia (n = 1), hungry bone syndrome (n = 3), and other causes (n = 2).

In 86.1% (n = 93) of patients, there was a previous diagnosis of hypoparathyroidism when they were first evaluated in the hospital, while in 13.9% (n = 15), the diagnosis was made in the follow-ups conducted at the institution. A total of 91.7% (n = 99) were women, with a mean age of 51.6 years (SD 15.6), and the most frequently identified etiology was postoperative (93.5%), followed by idiopathic (4.6%), cause not described (0.9%), and post-radiation (0.9%). It is worth noting that in cases of postoperative hypoparathyroidism, patients who were operated on by general surgeons as opposed to specialists in head and neck surgery could not be distinguished because this data was not available in most histories. The main comorbidities identified were hypothyroidism (88.9%), thyroid carcinoma (51.9%), arterial hypertension (42.6%), and dyslipidemia (21.3%). The patients’ baseline characteristics are described in Table 1 .

**Table 1:** Demographic characteristics

Variables	N = 108 (%)
Gender	
Female	99 (91.7%)
Male	9 (8.3%)
Age (years)	51.6 ± 15.6 (18-82)
Ethnicity	
Hispanic ancestry	107 (99.1%)
Others	1 (0.9%)
Etiology	
Postoperative	101 (93.5%)
Idiopathic	5 (4.6%)
Not identified	1 (0.9%)
Post radiation	1 (0.9%)
Comorbidities	
Hypothyroidism	96 (88.9%)
Thyroid cancer	56 (51.9%)
Arterial hypertension	46 (42.6%)
Dyslipidemia	23 (21.3%)
Diabetes mellitus	7 (6.5%)
Chronic kidney disease	21 (19.4%)
Coronary artery disease	4 (3.7%)

The information is presented as mean ± SD (range) for age, and percentages for the other variables.

Mean PTH was 6.9 pg/mL (SD 5.6, p25-p75, 2.5-11), initial calcium was 7.8 mg/dL (SD 1.8), and mean lowest calcium was 7.2 mg/dL (SD 1.5). The average phosphorus value was 5 mg/dL (SD 1.2). Only the last calciuria was known in 18.5% of the total cohort (20 patients) and the average was 237.4 mg/24 h (SD 48.08, p25-p75, 96-309). 25OHD levels were measured in just 44.4% of the cases (48 patients) and an average level of 35.7 ng/mL was found (SD 2.36, p25-p75 23-49). Average albumin was 4.12 mg/dL. The clinical manifestations of the disease were varied and included paresthesia, musculoskeletal compromise, renal disease, neurological and psychiatric features. Those features are better showed in Table 2 . There was an average of 0.7 emergency consultations, although the range was very wide (0 to 21).

**Table 2:** Clinical manifestations

Clinical manifestations	N = 108 (%)
Paresthesia	38 (35.2%)
Musculoskeletal compromise	30 (27.8%)
Concomitant psychiatric manifestation*	18 (16.7%)
Carpopedal spasm	17 (15.7%)
Trousseau's sign	14 (13%)
Seizures	10 (9.3%)
Chvostek’s sign	7 (6.5%)
Basal ganglia calcification	6 (5.5%)
Nephrolithiasis**	4 (3.7%)
Nephrocalcinosis***	3 (2.8%)

*The most frequently reported psychiatric condition was depression in 7.4% of the whole cohort.

**Only 18 (16.6%) patients had imaging of the central nervous system.

***Only 10.1% (n = 11) patients had renal studies.

In the total cohort, it was found that the presentation of the calcium used, was calcium carbonate in 78.1% of cases and calcium citrate in the remaining 21.9%. Likewise, a similar proportion was maintained when the groups of controlled (77.8% carbonate, 22.2% citrate) and uncontrolled (75% carbonate, 25% citrate) patients were evaluated. The average dose of elemental calcium in the last control was 1,164 mg/day (p25-p75, 480-1,440); calcitriol was 0.7 ug/day (p25-p75, 0.5-1.0), and 28% of the population were receiving thiazide diuretics (n = 30). However, 9.2% (n = 10) required more than 2,500 mg/day of elemental calcium and 2.7% (n = 7) received more 1.5 ug/day of calcitriol. It was possible to assess disease control in 89 patients (82%) who had multiple assessments over time, of which 45 of them (57.3%) had control of the disease.

When comparing the group of controlled patients against uncontrolled patients, the PTH level in the group of controlled patients was higher compared to the group of uncontrolled patients (9.2 *vs.* 5.1), requiring lower doses of elemental calcium and calcitriol. However, these differences were no statistically significant. Similarly, higher values of calcium were observed with values of phosphorus, and lower calcium/phosphorus product in the group of controlled patients versus the uncontrolled group ( Table 3 ).

**Table 3:** Biochemical results and doses of calcium and calcitriol discriminated by disease control

Variables	Controlled (n = 45)	Uncontrolled (n = 44)
PTH	8.9 (2.75-14.75) (n = 45)	3.55 (2.5-6.92) (n = 45)
Calcium*	7.9 (7.17-8.6) (n = 45)	8.0 (6.63-8.5) (n = 45)
Phosphorusα	4.4 (3.9-5.05) (n = 41)	5.2 (4.23-6.1) (n = 42)
25OHD	31.8 (21.75-44.25) (n = 15)	38.7 (27.6-52.8) (n = 16)
Calcium/phosphorus product	35.6 (31.48-38.7) (n = 41)	39.6 (34.3-43.5) (n = 42)
Dose of elemental calcium	720 (450-1230) (n = 45)	1,260 (480-1440) (n = 45)
Calcitriol dose	0.5 (0.25-1.0) (n = 42)	0.5 (0.5-1) (n = 43)

The information is presented as: median (p25-p75) for the evaluated variables. *Initial calcium value. PTH pg/mL, calcium onset mg/dL, phosphorus, mg/dL, 25OHD ng/mL, calcium/phosphorus product mg^2^/dL^2^, dose of elemental calcium mg, dose of calcitriol ug. α: p < 0.01. There were no significative differences in the other variables.

The median time of follow-up was 860 days (IQR 344-1712). 72.6% were from Medellin City and 27.2 outside Medellin. (0.9% without data). All patients had social security. 33.3% belonged to a subsidized system and 65.8% to a contributive system (0.9% without data)

There was no difference in the follow up time in patients with controlled disease or controlled disease (Controlled: median time 1157 95% IC 1033-1549 *vs.* Uncontrolled: median 856 95 IOC 856-1396 p = 0.756). There was no difference also regarding city (p = 0.661) or social security (p = 0.40).

Two patients (1.9%) who were receiving rhPTH were documented, which in this case was teriparatide. Both patients were women of Hispanic ancestry with postoperative hypoparathyroidism. One of the patients was 47 years old, had a serum PTH of 3.5 ng/mL, serum calcium of 4.77 mg/dL, serum phosphorus of 6.8 mg/dL (calcium/phosphorus product of 32.4 mg^2^/dL^2^) and received 1.0 µg/day of calcitriol and 3,150 mg/day of elemental calcium. The other patient was 27 years old, had a serum PTH of 6.2 ng/mL, serum calcium of 4.8 mg/dL, serum phosphorus of 7.0 mg/dL (calcium/phosphorus product of 30.7 mg^2^/dL^2^) and received 2.0 µg/day of calcitriol and 2,880 mg/day of elemental calcium. There was no data for 24-hour calciuria neither for neurologic complications of these two patients.

There were 23 patients (21%) who met the criteria for rhPTH use. In the group of uncontrolled patients (n = 44), 16.7% (n = 15) met some of the criteria, while in the group of controlled patients (n = 45), 3.12% (n = 3) had an indication for this treatment. The remaining 5 patients met the criteria for initiation of rhPTH, but there was no follow-up to establish whether or not there was control of the disease. Data from these patients is showed in Table 4 .

**Table 4:** Rate of subjects that meet each criterion for rhPTH

Indication for rhPTH	N = 23*
Dose of elemental calcium > 2,500 mg/day	10 (43.5%)
Dose of calcitriol > 1.5 µg/day	3 (13%)*
Calcium/Phosphorus product > 55 mg^2^/dL^2^	3 (13%)*
Hypercalciuria**	9 (39.1%)*
Nephrolithiasis	4 (17.4%)*
Nephrocalcinosis	3 (13%)

*Some patients met more than one of the criteria: all three patients who met the criteria for dose of calcitriol also met the criteria for dose of elemental calcium; one of the patients who met the criteria for Ca/P product also met the criteria for dose of elemental calcium; in the hypercalciuria group, one met the criteria for elemental calcium dose, two met the nephrocalcinosis criteria and one the nephrolithiasis criteria; one patient in the nephrolithiasis group also met the nephrocalcinosis criteria.

**Hypercalciuria is defined as >250 mg/24h in women and >300 mg/24h in men.

## DISCUSSION

A cohort of 108 patients with hypoparathyroidism was evaluated in a high complexity hospital in the city of Medellín. To the best of our knowledge, this is the only study of its kind so far in the Colombian territory. The results obtained confirm the main etiology as postoperative (93.5%), with other causes being infrequent, which is in accordance with studies conducted in American and European populations. However, the percentage of cases of postoperative etiology was much higher than that described in the literature, which ranges between 62 and 78% ( 3 , 4 , 8 , 18 - 20 ). These results can be explained by the measures used to prevent postoperative hypoparathyroidism; of which, the most widely supported in the literature ( 21 - 24 ) are preservation of the parathyroid glands through meticulous dissection, preservation of their blood flow, and even autotransplantation or autologous transplantation of the gland, procedures which must be implemented by an expert surgeon in the area, a condition of this procedure that is not applied at all hospital centers in Colombia.

The most frequent comorbidities were hypothyroidism and thyroid carcinoma, a completely expected result, provided that the primary reason for cervical surgery is thyroid gland surgery and it is described in the literature that up to 90% of cases of postoperative hypoparathyroidism are due to surgeries that involve this gland ( 18 ). The main reason for performing a thyroidectomy or hemithyroidectomy was thyroid carcinoma, according to what is described in the literature ( 3 ). As for the clinical manifestations of the disease, the most frequently described symptoms were paresthesia, followed by musculoskeletal involvement which occurred in about a third of patients, similar to what has been documented in the literature, where these manifestations can occur in between 24% and 54% of patients, depending on the region ( 25 , 26 ). Very low frequencies of Trousseau’s (13%) and Chvostek’s signs (6.5%) were found; however, an association analysis between its existence and serum calcium levels was not made, therefore, we cannot know for sure what its production is to predict the presence of hypocalcemia ( 8 , 27 - 30 ). Depression was the most frequently documented psychiatric manifestation in our study, consistent with the described increased risk of suffering from psychiatric disorders in patients with hypoparathyroidism. The study by Underbjerg and cols. ( 30 ) reported an HR of 1.99 (95% CI 1.14-3.46) for depression and bipolar disorder, which has also been documented in other observational studies ( 28 , 31 ). Regarding complications, it was found that about half of patients in whom calcium was measured in a 24-h urine test met the definition of hypercalciuria (n = 9), of which 33% (n = 3) had renal disease imaging where associated complications (nephrocalcinosis and nephrolithiasis) were fully documented. Although these results lack reliability as it was a small proportion of the study population who had a calciuria measurement in 24-h urine testing and imaging to evaluate renal complications, it is suggested that every patient with hypercalciuria in the follow-up should have renal imaging done. Of the patients, 11.1% had imaging of the central nervous system, where basal ganglia calcifications were found in 33.3%, strikingly in a much smaller proportion than is usually described in different observational studies, in which it ranges from 52%-74% of cases ( 17 , 27 , 28 , 32 , 33 ).

Approximately half of the patients had adequate disease control and to achieve this, and this group required a lower dose of elemental calcium and calcitriol when compared to the group of uncontrolled patients. Similarly, it was documented that 23 patients (21%) of the cohort had an indication for rhPTH treatment, of which the vast majority were patients who were in the uncontrolled group. The low doses of elemental calcium and calcitriol that these patients were receiving in the study are striking considering the doses that are usually described for these patients ( 1 ).

Different studies have been conducted for evaluating the use of rhPTH 1-84 (Natpara^®^) ( 14 , 34 , 35 ), which have shown its usefulness in the management of patients with hypoparathyroidism – promptly demonstrating the need for lower doses of calcium and calcitriol, improvement in bone health and also in quality of life ( 12 , 14 ). The results regarding the impact on hypercalciuria have not been clear, only in a study where an infusion pump protocol was used was a 60%-70% reduction in calciuria demonstrated. Regarding the use of PTH 1-34 (teriparatide), although this drug is not approved for the treatment of patients with hypoparathyroidism, there has also been an impact on the daily dose of calcium and calcitriol, as well as improvement in quality of life and bone health. For all the above reasons, the use of PTH, mainly rhPTH 1-84 (Natpara^®^), may be implemented as part of the treatment in those patients who persist with poor control of their disease or develop long-term complications, as stated in the international management guidelines ( 11 ). However, rhPTH 1-84 is not available in our region and teriparatide is only approved for use in patients with osteoporosis, which limits the use of this replacement in daily clinical practice.

### Limitations

This is a retrospective study with the limitations inherent to the loss of data and collection based on data written by the clinicians. there is no way to know if the study subjects are representative of the original population, in addition, patients were identified via ICD-10 codes, the sample may thus not represent the totality of patients with hypoparathyroidism treated at the hospital during the period of time studied. The main reason for non-inclusion was the absence of PTH levels in the medical records despite the fact that many of these patients already had a previous diagnosis of hypoparathyroidism and were receiving calcium, which forced a high number of patients with hypoparathyroidism to be excluded.

Due to the retrospective nature of the study, some important aspects of the population could not be established: no data was found on what type of specialist performed the intervention in cases of postoperative hypoparathyroidism (general surgery vs. head and neck surgery) and it was not possible to establish the duration of the disease before diagnosis.

There were other laboratory parameters such as calciuria, 25OHD levels, and diagnostic imaging, among others that were not performed in all study patients or were not recorded in the medical records. Only a small proportion of them had all the parameters established for adequate follow-up of patients with hypoparathyroidism, which limits the interpretation of these results. It should be mentioned that the calcium recorded during the study corresponds to the first measurement of calcium recorded in the clinical history and a compilation of all serum calcium values of each patient was not made, however, said parameter was taken into account when defining disease control. The measurement of serum magnesium was also not taken into account.

It was not possible to establish the time of progress during which the patients maintained control of the disease, this is explained by the retrospective nature of the study and the inability to follow-up with patients who did not have multiple assessments during the last year.

In conclusion, to the best of our knowledge, this is the first study on the clinical profile of Colombian patients with hypoparathyroidism that shows that a higher percentage of cases are secondary to surgical interventions, when compared with other centers in the world. With regard to medical management, the doses of calcium and calcitriol used were relatively low, and with this it is possible to achieve disease control in little more than half of cases; on the other hand, patients who did not achieve adequate control received a seemingly higher dose of the medications, which can be explained by the individual variation of the disease.

Only a minority of patients received PTH therapy as an adjuvant in the pharmacological management of the disease, despite the fact that a fifth of the cohort met the criteria for its use. It is possible that the low use of this medicine explains, or at least partially explains, the low level of control that was achieved in the total cohort and that more widespread use of this therapy can considerably improve the proportion of patients who achieve the criteria for the control of disease, without increasing the risk of chronic complications associated with hypoparathyroidism.
